# The Tumor-Educated-Macrophage Increase of Malignancy of Human Pancreatic Cancer Is Prevented by Zoledronic Acid

**DOI:** 10.1371/journal.pone.0103382

**Published:** 2014-08-12

**Authors:** Yukihiko Hiroshima, Ali Maawy, Mohamed K. Hassanein, Rhiana Menen, Masashi Momiyama, Takashi Murakami, Shinji Miwa, Mako Yamamoto, Fuminari Uehara, Shuya Yano, Ryutaro Mori, Ryusei Matsuyama, Takashi Chishima, Kuniya Tanaka, Yasushi Ichikawa, Michael Bouvet, Itaru Endo, Robert M. Hoffman

**Affiliations:** 1 AntiCancer, Inc., San Diego, California, United States of America; 2 Department of Surgery, University of California San Diego, San Diego, California, United States of America; 3 Yokohama City University Graduate School of Medicine, Yokohama, Japan; The University of Texas MD Anderson Cancer Center, United States of America

## Abstract

We previously defined macrophages harvested from the peritoneal cavity of nude mice with subcutaneous human pancreatic tumors as “tumor-educated-macrophages” (Edu) and macrophages harvested from mice without tumors as “naïve-macrophages” (Naïve), and demonstrated that Edu-macrophages promoted tumor growth and metastasis. In this study, Edu- and Naïve-macrophages were compared for their ability to enhance pancreatic cancer malignancy at the cellular level in vitro and in vivo. The inhibitory efficacy of Zoledronic acid (ZA) on Edu-macrophage-enhanced metastasis was also determined. XPA1 human pancreatic cancer cells in Gelfoam co-cultured with Edu-macrophages proliferated to a greater extent compared to XPA1 cells cultured with Naïve-macrophages (*P* = 0.014). XPA1 cells exposed to conditioned medium harvested from Edu culture significantly increased proliferation (*P* = 0.016) and had more migration stimulation capability (*P*<0.001) compared to cultured cancer cells treated with the conditioned medium from Naïve. The mitotic index of the XPA1 cells, expressing GFP in the nucleus and RFP in the cytoplasm, significantly increased in vivo in the presence of Edu- compared to Naïve-macrophages (*P* = 0.001). Zoledronic acid (ZA) killed both Edu and Naïve in vitro. Edu promoted tumor growth and metastasis in an orthotopic mouse model of the XPA1 human pancreatic cancer cell line. ZA reduced primary tumor growth (*P* = 0.006) and prevented metastasis (*P* = 0.025) promoted by Edu-macrophages. These results indicate that ZA inhibits enhanced primary tumor growth and metastasis of human pancreatic cancer induced by Edu-macrophages.

## Introduction

The tumor microenvironment (TME) plays a major role in determining tumor behavior. We have previously shown that stromal cells are necessary for metastasis to occur [1]. Another indication of the role of the TME in influencing tumor behavior is orthotopic implantation in mouse models of intact tumor tissue, including the entire tumor stroma, which results in a much higher metastatic rate compared to orthotopic injection of cancer cells alone, where metastasis is rare [2], [3].

Tumor-associated macrophages have been shown to correlate with poor prognosis in several studies [4], [5]. Macrophages may promote tumor progression by chronic inflammation, matrix remodeling, promotion of tumor-cell invasion, intravasation, angiogenesis, and seeding at distant sites [6]. Vascular cell adhesion molecule-1 (VCAM-1) promotes lung metastasis in breast cancer by tethering cancer cells to lung metastasis-associated macrophages [7], [8].

Our laboratory previously compared primary and metastatic tumor growth following the addition of either tumor-naïve-macrophages (Naïve) or macrophages previously exposed to pancreatic cancer in a mouse model, which were termed tumor-educated-macrophages (Edu) [8].

Our previous results suggest that macrophages influence tumors and tumors influence macrophages, and that Edu-macrophages promote tumor progression [8].

In the present study, Edu- and Naïve-macrophages were compared for their ability to promote malignancy at the cellular level in vitro and in vivo. The efficacy of Zoledronic acid (ZA) to inhibit Edu-macrophage-enhanced malignancy was also determined.

## Materials and Methods

### Cell line and culture conditions

The XPA1 human pancreatic cancer cell line was used in this study which was a kind gift from Dr. Anirban Maitra at Johns Hopkins University [6]–[16]. The XPA1 cell line was transformed to stably express GFP in the nucleus and RFP in the cytoplasm [9], [17], [18]. Cells were maintained in RPMI 1640 medium supplemented with 10% fetal bovine serum (FBS) and 2 mM glutamine (Gibco-BRL, Life Technologies Inc., Grand Island, NY). All media were supplemented with penicillin and streptomycin (Gibco-BRL). Cells were cultured at 37°C with 5% CO_2_ [19].

### Animals

NOD/SCID mice and athymic nude mice (*nu/nu*), 4–6 weeks, (AntiCancer Inc., San Diego, CA) were used. Transgenic nude C57/B6-GFP mice (AntiCancer, Inc., San Diego, CA). expressing green fluorescent protein (GFP) under the control of the chicken β-actin promoter and cytomegalovirus enhancer were also used [20]–[23]. Mice were kept in a barrier facility under HEPA filtration. Mice were fed with autoclaved laboratory rodent diet. All surgical procedures and imaging were performed with the animals anesthetized by intramuscular injection of 0.02 ml of a solution of 50% ketamine, 38% xylazine, and 12% acepromazine maleate. All animal studies were conducted with an AntiCancer Institutional Animal Care and Use Committee (IACUC)-protocol specifically approved for this study and in accordance with the principals and procedures outlined in the National Institute of Health Guide for the Care and Use of Animals under Assurance Number A3873-1.

### Subcutaneous tumor cell implantation

Human XPA1 dual-color pancreatic cancer cells were harvested by trypsinization and washed twice with serum-free medium. Cells (2×10^6^ in 100 µl serum-free media) were injected subcutaneously, within 30 min of harvesting, over the flanks in transgenic nude C57/B6-GFP mice or nontransgenic *nu/nu* mice between 4 and 6 weeks of age. Subcutaneous tumors were allowed to grow for 2–4 weeks until large enough for further experiments or subsequent orthotopic implantation.

### Real-time imaging of the interactions between host macrophages and cancer cells in live mice

**Figure 1:**
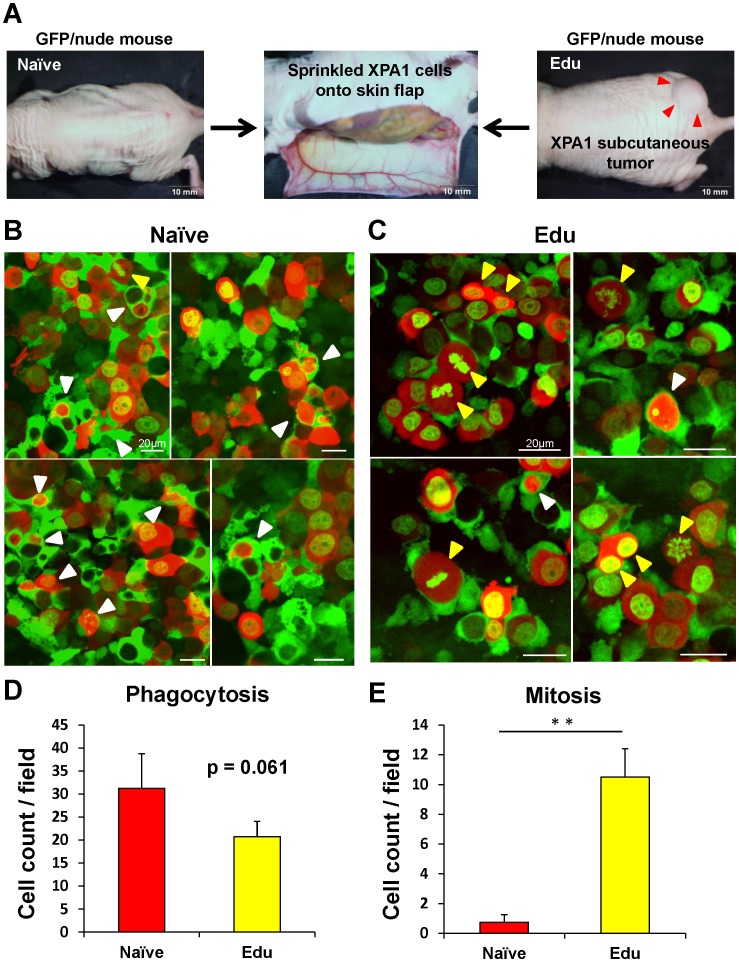
Imaging of the interactions between host macrophages and cancer cells in live mice. (**A**) Scheme of imaging the interactions between host macrophages and cancer cells in live mice. GFP nude mice with dual-color XPA1 subcutaneous tumors were the source of Edu-macrophages. GFP nude mice without tumors were the source of Naïve-macrophage. A skin-flap was spread and fixed on a flat stand. XPA1-GFP-RFP cells (1×10^6^ in 100 µl medium) were sprinkled over the surface of the skin–flap of mice. Twenty-four hours later, the inside surface of the skin-flap was directly observed with the FV1000 confocal microscope (Olympus). Scale bars: 10 mm. GFP host macrophages and dual-color XPA1 cancer cells were observed in Naïve-macrophage mice (**B**) and Edu-macrophage mice (**C**). Phagocytosed cancer cells (white arrowheads) and mitotic cancer cells (yellow arrowheads) were detected in both groups. The mean values of phagocytosized and mitotic cancer cells were calculated from four fields with a 20× magnification objective (**D and E**). Mitosis significantly increased in cancer cells in mice with Edu-macrophages compared to cancer cells in mice with Naïve-macrophages (*P* = 0.001). More phagocytosis of cancer cells tended to be detected in mice with Naïve-macrophages, (*P* = 0.061). Scale bars: 20 µm.

After the mice were anesthetized as described above, an arc-shaped incision was made in the abdominal skin, and then subcutaneous connective tissue was separated to free the skin flap without injuring the epigastric cranialis artery and vein [24]. The skin-flap was spread and fixed on a flat stand. XPA1-GFP-RFP cells (1×10^6^ in 100 µl medium) were sprinkled over the surface of the skin–flap of mice (Fig. 1A) [25]. Twenty-four hours later, the inside surface of the skin-flap was directly observed with the FV1000 confocal microscope (Olympus, Tokyo, Japan) with excitation from semiconductor lasers at 473 nm for GFP and 559 nm for RFP excitation. Fluorescence images were obtained using the 20×/1.0 XLUMPLFLN objective [26]. The number of phagocytosed and mitotic cancer cells were counted, and the mean number was calculated from five visual fields at 20× magnification.

### Macrophage harvest

Transgenic nude C57/B6-GFP mice with the dual-color XPA1-GFP-RFP subcutaneous tumors were the source for Edu-macrophages. Transgenic nude C57/B6-GFP mice without tumors were the source for Naïve-macrophages. After the mice were anesthetized as described above, 6 ml RPMI was injected into the intraperitoneal space of each mouse using a 10 ml syringe and the mice were gently agitated for 5 minutes. The syringe was then reinserted and all RPMI medium was removed and placed into a plastic petri dish. The dishes were then incubated at 37°C for 4 hours. RPMI medium was then removed and each plate was washed 10–15 times with 10 ml PBS, or until all red blood cells, fibroblasts, and other cellular debris were removed. Plates were visualized under an IX71 fluorescence microscope (Olympus, Tokyo, Japan) in order to confirm the presence of GFP-expressing macrophages. Macrophages were scraped from the petri dish, using a rubber spatula, collected with a wash of 10 ml PBS, and centrifuged at 850 rpm for 10 minutes. PBS was then removed and macrophages were resuspended in RPMI.

### Proliferation assay using 3D culture

**Figure 2:**
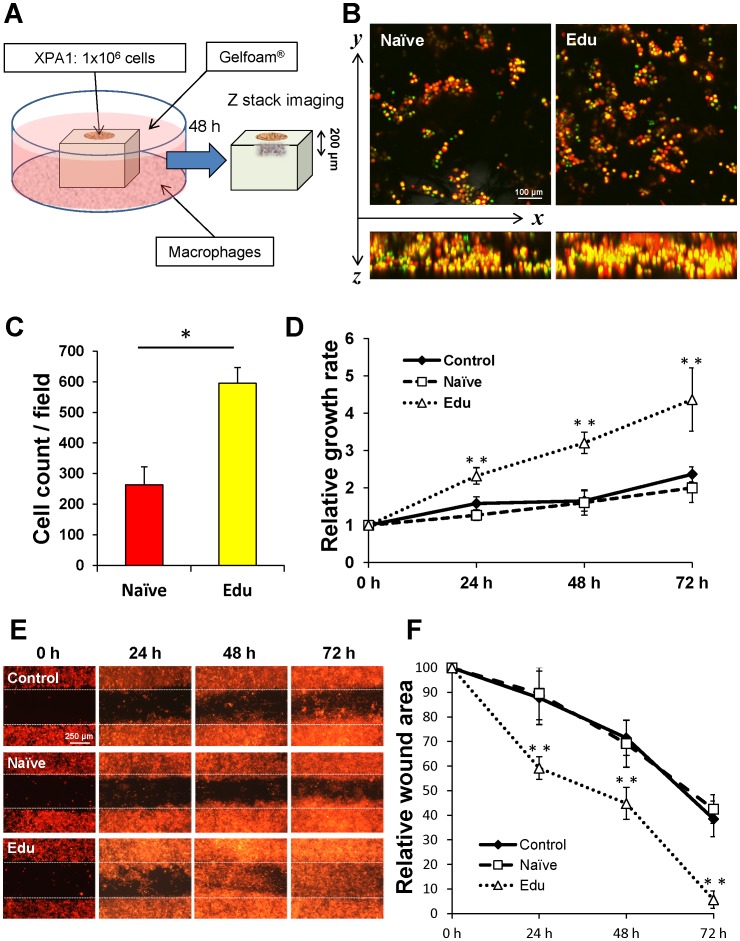
Conditioned medium from Edu-macrophages promotes pancreatic cancer cell proliferation and migration. (**A**) A schema of the separated co-culture system using Gelfoam. Naïve- or Edu-macrophages were co-cultured with dual-color XPA1 GFP-RFP cancer cells, but they were separated from each other by Gelfoam. Forty-eight hours later, images were obtained from the surface to 200 µm depth, every 1 µm, and were stacked along the Z axis. (**B**) Representative Z-stack image using a 20× magnification objective. (**C**) The mean number of cancer cells was calculated from three fields of Z-stack images with the 20× magnification objective. The number of cancer cells treated with Edu-macrophages increased compared to the number of cancer cells treated with Naïve-macrophages (*P* = 0.014). (**D**) MTS proliferation assay. Conditioned medium was harvested from each type of macrophage cultured in RPMI without serum for 72 h. The culture medium from the cancer cells was removed from the wells and the conditioned medium was added. Viable cancer cell numbers are indicated with the MTS assay at various time points. The line graph indicates the relative proliferation rate of each group. The proliferation rate of Edu-macrophage-condition-medium-treated cancer cells was significantly higher than control or Naïve-macrophage-conditioned-medium-treated cancer cells. Black diamonds: Naïve; white square: Edu, white triangle: control group. * *P*<0.05, ** *P*<0.01 (vs. control group). (**E**) Wounded areas were measured for 72 hours after scratching the monolayer of cancer cells and treatment with the conditioned medium from each type of macrophage. (**F**) Edu-macrophage-conditioned medium-treated cells covered a significantly larger wound area than other groups at every time point. Charts are plots of wounded areas of each group measured with ImageJ of three random areas in 3 days. Data from three repeat experiments are presented as mean ± SD (n = 5). * *P*<0.05, ** *P*<0.01 (vs. control group).

Naïve- or Edu-macrophages were co-cultured with dual-color XPA1-GFP-RFP cancer cells, but they were separated from each other by Gelfoam (Pharmacia & Upjohn Company, MI, USA) (Fig. 2A). Forty-eight hours later, images were obtained from the surface to 200 µm depth, every 1 µm, with the FV1000 confocal microscope. The images were stacked along the Z axis, and the mean number of cancer cells was calculated from three visual fields at 20× magnification.

### MTS proliferation assay

XPA1-GFP-RFP cells in the exponential growth phase were trypsinized to yield a cell suspension and seeded onto 96-well plates (1×10^4^ cells/well) in triplicate. Conditioned medium was harvested from each type of macrophages cultured in RPMI 1640 with 10% FBS for 72 h. The culture medium was removed from the wells with XPAI-GFP-RFP cells, and the conditioned medium was added after the cells were allowed to adhere for 24 h. After the plates were incubated at 37°C in 5% CO_2_, the MTS assay was performed at various time points (0–72 h) using the Cell Titer 96 assay (Promega, Madison, WI, USA) according to the manufacturer's instructions.

### Wound healing assay

Cells were grown in 24-well plates in 500 µl medium per well until confluence was reached. A wound was made by scratching the cells with a 10 µl pipette tip in PBS, followed by replacement with conditioned medium which was harvested from each macrophage type cultured in RPMI 1640 with 10% FBS for 72 h. The control group received the same volume of fresh culture medium without serum. The wounded monolayer was photomicrographed with the IX71 fluorescence microscope at different time points (0–72 h) after being scratched. Cell migration was assessed by measuring gap sizes in multiple fields using Image J (National Institute of Mental Health, Bethesda, MD).

### Cytotoxicity assay

Naïve- or Edu-macrophages were seeded onto 6-well plates (2×10^5^ cells/well). Zoledronic acid (ZA) (Novartis Pharmaceuticals Corporation, NJ, USA) was added to each well after the cells were allowed to adhere for 24 h. The final concentrations of ZA were as follows: 0, 10, 50 and 100 µM. After 48 h, the ZA-containing medium was removed and replaced with growth medium for 24 h. After each well was washed 5 times with 3 ml PBS, the number of GFP-expressing macrophages was counted with the IX71 fluorescence microscope. The mean macrophage number was calculated from five visual fields at 10× magnification. All measurements were carried out in triplicate.

### Orthotopic tumor implantation

A small 6- to 10-mm transverse incision was made on the left flank of the mouse through the skin and peritoneum. The tail of the pancreas was exposed through this incision and a single tumor fragment (3-mm^3^) from a XPA1 dual-color subcutaneous tumor was sutured to the tail of the pancreas using 8-0 nylon surgical sutures (Ethilon; Ethicon Inc., NJ, USA). Upon completion, the tail of the pancreas was returned to the abdomen, and the incision was closed in one layer using 6-0 nylon surgical sutures (Ethilon) [2], [27]–[29].

### ZA treatment of the XPA1 orthotopic nude mouse model

Nude mice were orthotopically implanted with XPA1-GFP-RFP cells as described above. The mice were treated in the following groups: (1) saline (vehicle/control), (2) Naïve- macrophages (3) Edu-macrophages, (4) Edu-macrophages + ZA. Macrophages (1×10^6^ cells in 200 µl PBS) were injected ip weekly as previously described [8]. ZA was subcutaneously injected daily from day 21 after tumor implantation for 4 weeks. Each treatment arm involved 8 tumor-bearing mice. No significant effects on body weight, morbidity, or severe toxicity were observed in any treatment arm. Animals underwent laparotomy at 7 weeks, and both primary tumors and metastases were imaged using the OV100 variable magnification Small Animal Imaging System (Olympus, Tokyo, Japan) [24] and weighed and harvested for analysis.

### Data processing and statistical analysis

PASW Statistics 18.0 (SPSS, Inc) was used for all statistical analyses. The Student's t-test was used to compare continuous variables between two groups. Analysis of variance models were used to compare multiple groups. A p value of 0.05 was considered statistically significant for all comparisons.

## Results and Discussion

### Edu-macrophages stimulate cancer-cell mitosis in vivo

Dual-color XPA1-GFP-RFP cells (1×10^6^) were sprinkled over the surface of a mouse skin-flap. Twenty-four hours later, the inside surface of the skin-flap was directly observed with the FV1000 confocal microscope (Olympus) (Fig. 1A). GFP-expressing host macrophages and dual-color XPA1 cancer cells were observed in Naïve-macrophage mice (Fig. 1B) and Edu-macrophage mice (Fig. 1C). Cancer-cell mitosis was significantly greater in cancer cells in Edu-macrophage mice compared to cancer cells in Naïve-macrophage mice (*P* = 0.001) (Fig. 1E). Phagocytosis of cancer cells tended to be greater in Naïve-macrophage mice compared to Edu-macrophage mice (*P* = 0.061) (Fig. 1D).

### Edu-macrophages stimulate proliferation of cancer cells *in vitro*

Naïve- or Edu-macrophage were cultured with dual-color XPA1 cancer cells separated from each other by Gelfoam (Fig. 2A). Forty-eight hours later, confocal microscopy images were obtained from the surface to 200 µm depth every 1 µm and were stacked along the Z axis (Fig. 2B). Edu-macrophage-treated cancer cells proliferated to a greater extent compared to Naïve-macrophage-treated cancer cells (*P* = 0.014) (Fig. 2C). Conditioned medium from Edu-macrophages stimulated the proliferation of cancer cells compared to control untreated cancer cells (24 h; *P* = 0.001, 48 h; *P* = 0.003, 72 h; *P* = 0.012, respectively). In contrast, there was no significant difference between conditioned medium from Naïve-macrophages and fresh medium on proliferation of XPA1 cells (Fig. 2D).

Edu-macrophage-conditioned-medium-treated cancer cells covered a significantly larger wound area than untreated control cells at every time point (24 h; *P* = 0.008, 48 h; *P* = 0.002, 72 h; *P*<0.001, respectively) (Fig. 2E and 2F).

### ZA inhibits XPA1 pancreatic tumor progression induced by Edu-macrophages

**Figure 3:**
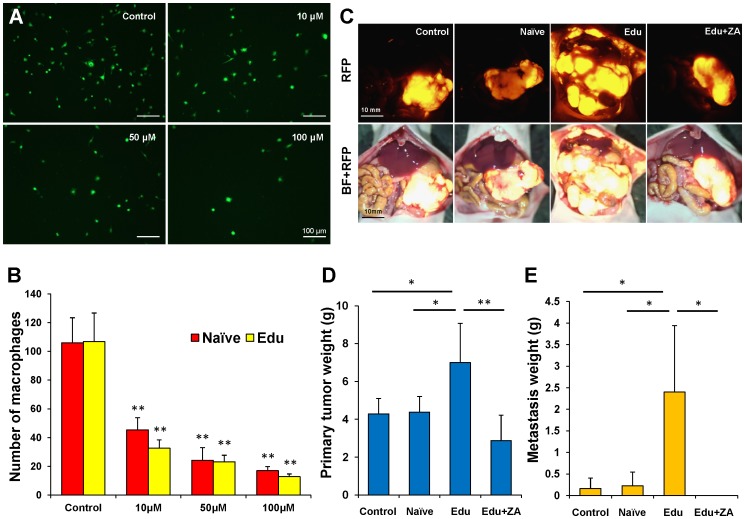
ZA inhibits Edu-macrophages and tumor progression induced by Edu macrophages in the orthotopic XPAI pancreatic cancer mouse model. (**A**) Representative fluorescence images of Edu-macrophages after ZA treatment. Scale bars: 10 µm. (**B**) Bar graphs of the number of Naïve- or Edu-macrophages after ZA treatment in vitro. The numbers of both Naïve- and Edu-macrophages treated with ZA were significantly reduced at every dose. ZA killed both Naïve and Edu in a dose-dependent manner. ** *P*<0.01 (vs. control group). (**C**) Intravital imaging of XPA1-RFP tumor-bearing mice at the termination of the experiment. Scale bars: 10 mm. (**D**) The primary tumor weight of Edu-macrophage-treated mice were significantly increased compared to control or Naïve-macrophage-treated mice (control: *P* = 0.026; Naïve: *P* = 0.03, respectively). The primary tumor weight of Edu-macrophage + ZA-treated mice were significantly decreased compared to Edu-macrophage-treated mice (*P* = 0.006). * *P*<0.05, ** *P*<0.01. (**E**) The metastasis weight of Edu-macrophage-treated mice were significantly increased compared to control or Naïve-macrophage-treated mice (control; *P* = 0.012, Naïve; *P* = 0.015, respectively). No metastasis was detected in Edu + ZA-treated mice. There was a significant difference between Edu and Edu + ZA-treated mice (*P* = 0.025). * *P*<0.05.

Edu-macrophages promoted primary tumor growth compared to control and Naïve- macrophage (control; *P* = 0.026, Naïve; *P* = 0.03). Edu-macrophages also promoted metastasis compared to control and Naïve-macrophages (control; *P* = 0.012, Naïve; *P* = 0.015) (Fig. 3C–E). ZA has been shown to kill macrophages and prevent metastasis [30], [31]. The number of both Naïve- and Edu-macrophages were significantly reduced by ZA at every dose in vitro (Fig. 3A and 3B). In an orthotopic mouse model of pancreatic cancer, ZA significantly suppressed Edu-macrophage, stimulated tumor growth, and prevented metastasis compared to the mice treated with only Edu-macrophages (primary tumor; *P* = 0.006, metastasis; *P* = 0.025) (Fig. 3C–E).

In our previous study, GFP-expressing macrophages from GFP transgenic nude mice with a subcutaneous BxPC3-RFP human pancreatic tumor were used as a source of Edu-macrophages and compared to Naïve-macrophages from the transgenic GFP nude mice without tumors. When the Edu- or Naïve-macrophages were then implanted into nude mice with BxPC-3-RFP growing orthotopically, the Edu-macrophages stimulated tumor growth and metastasis to a greater degree than Naïve-macrophages [8].

In another previous study, human peripheral-blood mononuclear cells were exposed to conditioned medium from BxPC-3 human pancreatic cancer cells, in normal or high glucose levels. The pancreatic cancer cells educated the macrophages to be more invasive in vitro, which was further enhanced by hyperglycaemia [32].

In another previous study, ZA reduced the macrophage-induced invasiveness of breast cancer cells. ZA affected macrophages but not the cancer cells [33].

Another previous study showed that both peritoneal and breast-tumor associated macrophages rapidly took up ZA in vivo, further suggesting macrophages are a target of the anti-tumor efficacy of ZA [34].

In another previous study, ZA directly caused apoptosis on pancreatic carcinoma cells and inhibited their invasiveness and as well as making the pancreatic cancer cells more vulnerable to T-cell attack [35].

The present study used subcellular imaging techniques we have previously developed [36] to directly image that Edu-macrophages have reduced phagocytosis ability and can directly simulate mitosis of the cancer cells, as visualized in real time. Unlike previous studies, evaluating the efficacy of ZA, the present report used an orthotopic metastatic mouse model to demonstrate the effect of ZA on the stimulation by Edu-macrophages on metastasis as well as on the primary tumor. In the present study, the macrophages were isolated from the peritoneal cavity from tumor-bearing or control mice, far from the tumor and administered systemically. The results indicated that tumors had distant affect on macrophages and vice versa and that ZA could systemically inhibit such effects.

ZA reduced Edu-stimulated primary tumor growth and metastasis to approximately control values suggesting the major ZA effect was on the Edu-macrophages, although a relatively minor direct inhibitory effect on the tumor itself by ZA cannot be ruled out as described in a previous study [35]. ZA has been previously reported to have greater macrophage killing efficacy than other bisphosphonates and was therefore chosen for the present study [37].

The present study indicated that Edu-macrophages promote malignancy. This was shown by the stimulatory effect of Edu-macrophages on cancer-cell mitosis and subsequent proliferation as well as on metastasis. As we stated previously [8], tumors can affect macrophages which in turn can affect tumors. Tumor progression induced by Edu-macrophages was inhibited by ZA, which inhibited Edu-macrophage-enhanced primary tumor growth and prevented metastasis. The mechanisms by which Edu-macrophages stimulate cancer-cell proliferation and progression will be the subject of a future study.
